# Update zum perioperativen Umgang mit kardial implantierten elektronischen Geräten

**DOI:** 10.1007/s00101-026-01657-3

**Published:** 2026-03-11

**Authors:** Fiona Uhor, Andreas Haeberlin, Jürgen Knapp

**Affiliations:** 1 Klinik für Anästhesiologie und Schmerzmedizin, Universitätsspital Bern, Inselspital, Universität Bern, Freiburgstrasse, 3010 Bern, Schweiz; 2 Klinik für Kardiologie, Universitätsspital Bern, Inselspital, Universität Bern, Bern, Schweiz

**Keywords:** Kabellose Schrittmacher, Implantierbare Kardioverter-Defibrillatoren, Kardiale Resynchronisationstherapie, Conduction System Pacing, Elektromagnetische Interferenzen, Leadless pacemaker, Implantable cardioverter defibrillators, Cardiac resynchronization therapy, Conduction system pacing, Electromagnetic interference

## Abstract

Das Fachgebiet der kardial implantierbaren elektronischen Geräte (CIED) hat in den vergangenen Jahren einen kontinuierlichen Zustrom neuartiger Technologien erfahren. Herzschrittmacher werden zunehmend als kabellose Systeme implantiert, die biventrikuläre kardiale Resynchronisationstherapie (CRT) ist fest etabliert, und subkutane und extravaskuläre implantierbare Kardioverter-Defibrillatoren (S-ICD, EV-ICD) stehen inzwischen ebenfalls zur Verfügung. Darüber hinaus widerspiegeln Innovationen wie die direkte Stimulation des kardialen Reizleitungssystems (sog. Conduction-system-pacing-Systeme) sowie sog. Loop Recorder mit Abmessungen kleiner als ein USB-Stick das bemerkenswerte Tempo des technischen Fortschritts in diesem Bereich. Für Anästhesiefachkräfte sind diese Entwicklungen besonders relevant. Die wachsende Prävalenz von Patientinnen und Patienten mit CIED, die sich nichtkardiochirurgischen Eingriffen unterziehen, macht ein fundiertes Verständnis der Gerätefunktionalität, perioperativer Managementstrategien und potenzieller Wechselwirkungen mit anästhesiologischen Verfahren erforderlich. Dieser Artikel bietet einen Überblick über die bedeutendsten technologischen Fortschritte im Bereich der CIED und stellt praxisorientierte Empfehlungen vor, die eine sichere und effektive Anwendung im anästhesiologischen Alltag unterstützen.

**Zusatzmaterial online:**

Die Online-Version dieses Beitrags (https://doi.org/10.1007/s00101-026-01657-3) enthält den „Algorithmus zum Perioperativem Management bei ICD und Herzschrittmachern“ als PDF zum Download. Bitte scannen Sie den QR-Code.

## Abstract (en)

The discipline of cardiac implantable electronic devices (CIED) has undergone numerous technical innovations in recent years. Leadless pacemaker systems are increasingly being implanted, biventricular cardiac resynchronization therapy (CRT) is firmly established and subcutaneous or extravascular implantable cardioverter defibrillators (S-ICD, EV-ICD) have become available. Moreover, advances such as direct stimulation of the cardiac conduction system with conduction system pacing systems and loop recorders with dimensions smaller than a USB stick illustrate the remarkable pace of technological innovation. For anesthesiology personnel these innovations have particular clinical relevance. The rising prevalence of patients with CIED undergoing noncardiac surgery underscores the need for a thorough understanding of device functionality, perioperative management strategies and potential interactions with anesthetic techniques. This article provides an overview of the most significant technological advances in the field of CIED and outlines practical recommendations to support the safe and effective integration into daily anesthesiological practice.

## Lernziele

Nach der Lektüre dieses Beitrags

- kennen Sie die wichtigen Schritte in der präoperativen Diagnostik bei Trägerinnen und Trägern von kardial implantierbaren elektronischen Geräten (CIED).
- können Sie die anästhesiologisch relevanten Unterschiede zwischen transvenösen und kabellosen Herzschrittmachern benennen.
- identifizieren Sie die Risikofaktoren für elektromagnetische Interferenzen zuverlässig.
- wissen Sie, was bei CIED-Trägerinnen und -Trägern bei der intra- und postoperativen Betreuung beachtet werden muss.
- kennen Sie Notfallmaßnahmen bei Fehlfunktionen des Schrittmachers.

## Einleitung

Die Zahl der Schrittmacher-Implantationen in Europa zeigt seit Jahren einen kontinuierlichen Anstieg, während jene der implantierbaren Kardioverter-Defibrillatoren (ICD) nahezu unverändert bleibt. Im Jahr 2022 wurden gesamteuropäisch durchschnittlich 866 Schrittmacher pro Million Einwohnerinnen und Einwohner sowie 120 ICD pro Million Einwohnerinnen und Einwohner neu implantiert [1].

Obwohl die Mehrheit der **Neuimplantationen**

Neuimplantationen

kardialer implantierbarer elektronischer Geräte (cardiac implantable electronic devices, CIED) nach wie vor auf konventionelle, transvenöse Systeme entfällt, zeigt sich ein exponentieller Anstieg in der Nutzung moderner Geräte, die kabellos funktionieren und minimalinvasiv implantiert werden können. Darüber hinaus besteht inzwischen die Möglichkeit, durch moderne Schrittmacher und ICD gezielt das kardiale Reizleitungssystem zu stimulieren und so eine physiologischere Stimulation des Herzens zu erzielen. Diese Weiterentwicklung im kardiologischen Bereich hat unmittelbare Konsequenzen für das perioperative Management der Patientinnen und Patienten, die mit diesen modernen Geräten ausgestattet sind. Anästhesiologisches Fachpersonal ist daher gefordert, über grundlegende Kenntnisse zu einer Vielzahl von CIED zu verfügen und sich kontinuierlich mit den technischen Neuerungen vertraut zu machen.

Der vorliegende Artikel soll bestehendes Wissen zum perioperativen Management von Patientinnen und Patienten mit herkömmlichen Systemen auffrischen und auf anästhesiologisch relevante Aspekte in der Behandlung von Patientinnen und Patienten mit modernen Devices wie kabellosen Schrittmachern, subkutanen und extravaskulären ICD und **Conduction System Pacing**

Conduction System Pacing

eingehen. Auf eine detaillierte Darstellung der Grundlagen der Schrittmachertechnologie wird dabei verzichtet.

### Fallbeispiel

Ein 77-jähriger Patient stellt sich drei Wochen vor einer elektiven Ileostoma-Rückverlegung nach Sigmavolvulus in der anästhesiologischen Sprechstunde vor. In der Anamnese findet sich neben einer arteriellen Hypertonie auch ein AV-Block III°, weshalb ein Jahr zuvor ein Herzschrittmacher implantiert wurde. Anhand des Schrittmacher-Ausweises kann der genaue Gerätetyp eruiert werden. Es handelt sich dabei um einen transvenösen Einkammerschrittmacher, dessen Aggregat pektoral links gut palpabel ist. Aufgrund der Entfernung des Operationsgebietes zum Schrittmacheraggregat wird beschlossen, präoperativ keine Umprogrammierung durchzuführen.

### Infobox 1 Nerd-Wissen.

*Warum werden Schrittmacher und ICD meist über die linke V. subclavia implantiert?*

- *Links ist bei der Mehrzahl der Patientinnen und Patienten die nicht-dominante* *Seite*
- *Der anatomische Verlauf der Vene ist gerader als rechtsseitig*
- *Von links eingelegte ICD zeigen niedrigere Defibrillationsschwellen als von rechts kommende*

Im Rahmen der präoperativen Abklärung bei Patientinnen und Patienten mit **CIED**

CIED

sind folgende Fragen zu klären und das entsprechende Prozedere für die intraoperativ betreuenden Kolleginnen und Kollegen zu dokumentieren:

- Welche Art von CIED ist implantiert?
- Ist die Patientin oder der Patient „Schrittmacher-abhängig“ (d. h., besteht ohne Stimulation durch das Gerät kein suffizienter Eigenrhythmus)?
- Ist das Gerät während der geplanten Operation für das betreuende Anästhesie-Team zugänglich?
- Wie reagiert das Gerät bei Auflage eines Magneten?

### Merke

Entscheidend zu Beginn der präoperativen Abklärung ist die Frage, ob es sich beim CIED um einen Schrittmacher oder einen Defibrillator handelt. So kann das weitere Vorgehen klar und einfach definiert werden.

## Transvenöse und kabellose Herzschrittmacher

Die Evidenzbasis sowie die klinische Erfahrung mit *konventionellen, transvenösen* Schrittmachern (Systemen mit Aggregat und Sonden) stützen sich auf jahrzehntelangen praktischen Einsatz. Ein wesentlicher Vorteil dieser Systeme liegt in der präzisen Anpassbarkeit wichtiger Parameter wie der **Rate Response**

Rate Response

(Anpassung der Stimulationsfrequenz an den aktuellen körperlichen Bedarf), der atrioventrikulären Überleitungszeit, Refraktärzeiten sowie in der zuverlässigen Speicherung von spontan aufgetretenen Arrhythmien. Der Anteil *kabelloser *Systeme beträgt zum aktuellen Zeitpunkt ca. 8 % aller implantierten Schrittmacher, wobei sowohl die Implantationsraten als auch der Marktanteil eine deutliche Zunahme verzeichnen, sodass zukünftig immer mehr Patienten mit solchen Geräten anästhesiologisch betreut werden [2, 3].

Erstmals wurde ein kabelloser intrakardialer Schrittmacher im Jahr 2012 einem Patienten implantiert. Im Unterschied zu den transvenösen Systemen erfolgt die Implantation direkt in den rechten Ventrikel, ohne die Notwendigkeit transvenöser Sonden oder einer pektoral angelegten **Generatortasche**

**Abb. 1:**
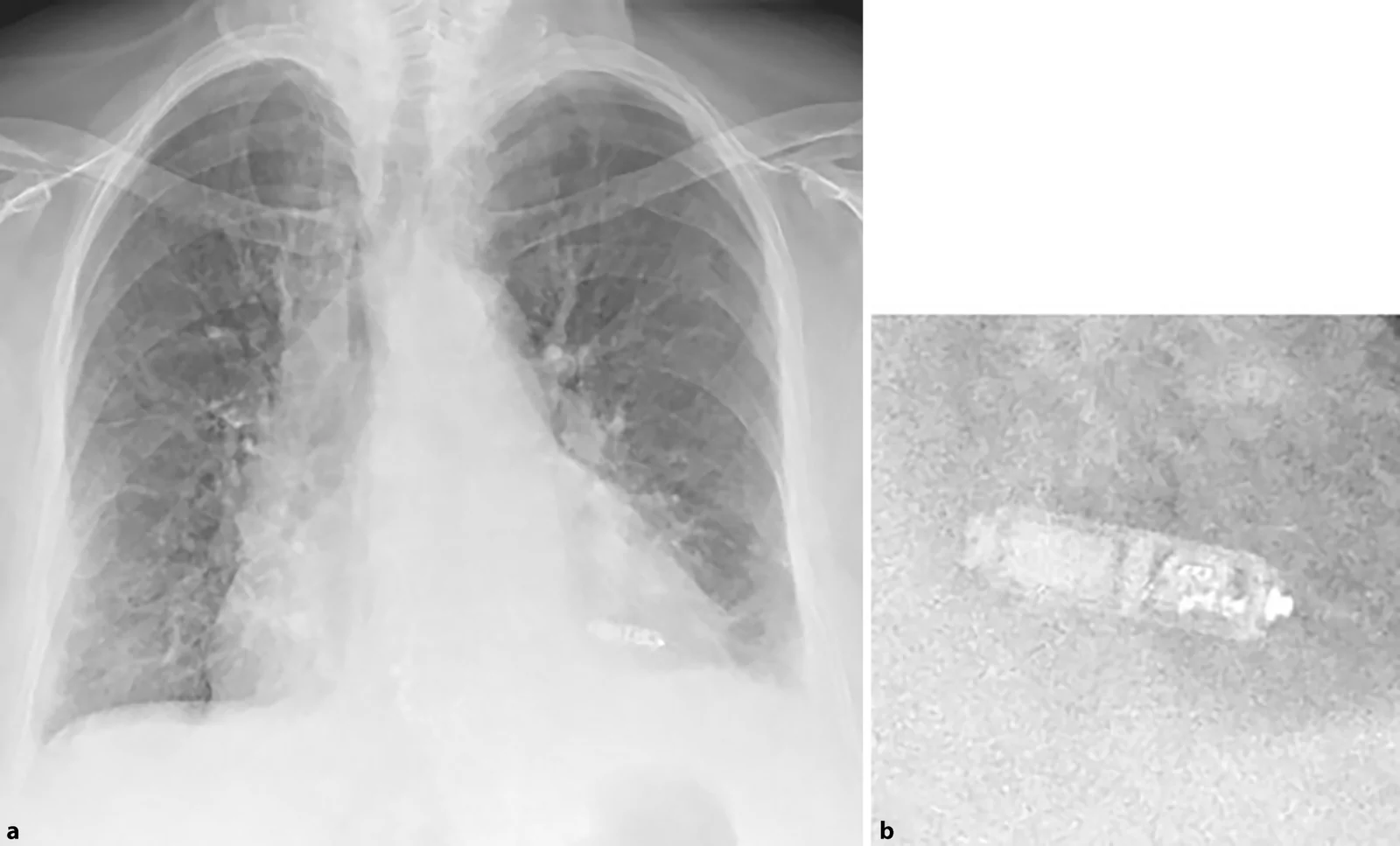
Darstellung eines kabellosen Einkammerschrittmachers im rechten Ventrikel. **a** p.-a.-Röntgenaufnahme, **b** Nachvergrößerung [7]

Generatortasche

(Abb. 1). Hierdurch lassen sich die typischen, mit Sonden assoziierten, Komplikationen vermeiden (Tab. 1). Gleichwohl können Dislokationen auftreten oder kann es während der Implantation zu einer Perforation des Myokards kommen. Obwohl aktuell noch keine prospektiven randomisierten kontrollierten Studien vorliegen, deuten bisherige Erfahrungen auf eine deutlich reduzierte Komplikationsrate bei kabellosen Systemen hin (3 % bei kabellosen vs. 7 % bei transvenösen Systemen), bei jedoch schwerwiegenderen Komplikationen [4]. Eine besondere Limitation stellt jedoch der Umgang mit einer Erschöpfung des Aggregats dar, da in diesem Fall kein einfacher Austausch über die **subkutane Tasche**

subkutane Tasche

möglich ist. Stattdessen muss ein neues System implantiert werden, während das inaktive Aggregat in der Regel im Ventrikel verbleibt oder mit speziellen Kathetern extrahiert werden muss [5, 6].

**Tab. 1:** Häufigste Komplikationen transvenöser im Vergleich zu kabellosen Schrittmachersystemen [8, 9].

Komplikationen transvenöser Systeme	Komplikationen kabelloser Systeme
Sondenbruch (1–12 %)	Dislokation des Gerätes (1–3 %)
Dislokation (4–8 %)	Perikarderguss (1,5 %)
Pneumothorax (3–4 %)	Perforation (1–1,5 %)
Isolationsdefekte (1–4 %)	Lokale Komplikationen in der Leistenregion (0,3–2 %)
Infektion (1,5 %)	
Venöser Verschluss (0–2 %)	
Perforation (0,4 %)	
Perikarderguss (0,35 %)	
Endokarditis (0,3 %)	

Die aktuellen Leitlinien der European Society of Cardiology (ESC) empfehlen den Einsatz kabelloser Schrittmacher daher insbesondere in Situationen, in denen kein transvenöser Zugang über die oberen Extremitäten zur Verfügung steht (z. B. bilaterales venöses **Thoracic-Outlet-Syndrom**

Thoracic-Outlet-Syndrom

oder chronische Obstruktion der V. cava superior) oder ein erhöhtes Infektionsrisiko besteht (z. B. stattgehabte CIED-Infektion oder Dialyse-Patientinnen und -Patienten). Darüber hinaus wird eine differenzierte Berücksichtigung von Alter und Lebenserwartung der Patientinnen und Patienten ausdrücklich hervorgehoben. Bei einer prognostizierten Lebenserwartung von mehr als 20 Jahren sind kabellose Systeme aufgrund der begrenzten **Aggregatlaufzeit**

Aggregatlaufzeit

nur in Ausnahmesituationen zu empfehlen [10].

## Einkammer- und Zweikammersysteme

Sowohl transvenöse als auch kabellose Schrittmacher existieren in Einkammer- und Zweikammervarianten. Einkammersysteme, die meist ausschließlich den rechten Ventrikel (RV) stimulieren (aber auch im rechten Atrium (RA) implantiert werden können), gewährleisten jedoch keine physiologische Erregungsleitung mit atrioventrikulärer Synchronisation. Der Einsatz des transvenösen Einkammerschrittmachers gilt daher heute weitgehend als obsolet, ausser bei Patientinnen und Patienten im permanenten Vorhofflimmern ohne Möglichkeit einer atrioventrikulären Synchronisation.

**Kabellose Systeme**

Kabellose Systeme

waren aufgrund technischer Restriktionen lange Zeit nur als Einkammerversion verfügbar. Seit 2023 stehen erstmals **kabellose Zweikammersysteme**

kabellose Zweikammersysteme

zur Verfügung, die eine Stimulation sowohl im RA als auch im RV erlauben. Die einzelnen Module kommunizieren hierbei über „conductive communication“ durch das Blut und Myokard miteinander [11].

### Merke

Kabellose Schrittmacher vermeiden sondenassoziierte Komplikationen und zeigen in bisherigen Registerstudien eine geringere Komplikationsrate.

## Systeme zur kardialen Resynchronisationstherapie (Dreikammerschrittmacher/ICD)

Bei Patientinnen und Patienten mit fortgeschrittener Herzinsuffizienz (Ejektionsfraktion (EF) < 35 %) und ventrikulärer Dyssynchronie (welche sich im EKG meist als Linksschenkelblock manifestiert) kann durch eine biventrikuläre, synchronisierte Stimulation des RV und des linken Ventrikels (LV) eine Verbesserung der EF sowie des Herzzeitvolumens (HZV) erzielt werden (sog. **kardiale Resynchronisationstherapie**

kardiale Resynchronisationstherapie,

CRT) [12]. Hierzu wird je eine Sonde im RA und im RV platziert, während eine dritte Sonde über den Sinus coronarius zum lateralen Epikard des LV geführt wird. Von dort aus erfolgt die Erregung des LV direkt übers Arbeitsmyokard. Wichtig bei Patienten mit Geräten zur CRT ist die klare Unterscheidung zwischen einem solchen biventrikulären Schrittmacher (CRT-P) und einer CRT mit zusätzlicher Defibrillator-Funktion (CRT-D).

### Merke

Da es sich bei Trägerinnen und Trägern einer CRT meist um kardial schwer vorerkrankte Patientinnen und Patienten handelt, sollte intraoperativ nach Nutzen-Risiko-Abwägung die Indikation zur invasiven arteriellen Blutdruckmessung großzügig gestellt werden.

## Conduction System Pacing

Eine therapeutische Alternative zu CRT und auch konventionellen transvenösen Schrittmachern/ICD stellt das **Conduction System Pacing**

Conduction System Pacing

(CSP) dar. Hierbei erfolgt die elektrische Stimulation des Herzens unmittelbar im kardialen Erregungsleitungssystem. Das Schrittmacher-Aggregat wird wie bei anderen Schrittmacher-Systemen subkutan pektoral implantiert. Die beiden Hauptvarianten des CSP sind das **His-Bündel-Pacing**

His-Bündel-Pacing

(HBP) sowie die Stimulation im Bereich des linken Tawara-Schenkels (LBBAP – Left Bundle Branch Area Pacing). Ziel dieser Technik ist eine möglichst physiologische ventrikuläre Aktivierung, welche langfristig mit einer geringeren Wahrscheinlichkeit einer schrittmacherinduzierten Kardiomyopathie und damit assoziierter Morbidität und Mortalität einhergeht [13, 14]. Auch im CSP kann, analog zum CRT‑D, eine ICD-Funktion integriert sein.

### Merke

Conduction System Pacing stimuliert direkt das kardiale Erregungsleitungssystem, um eine möglichst physiologische Ventrikelaktivierung zu erreichen.

## Transvenöse implantierbare Kardioverter-Defibrillatoren

**Abb. 2:**
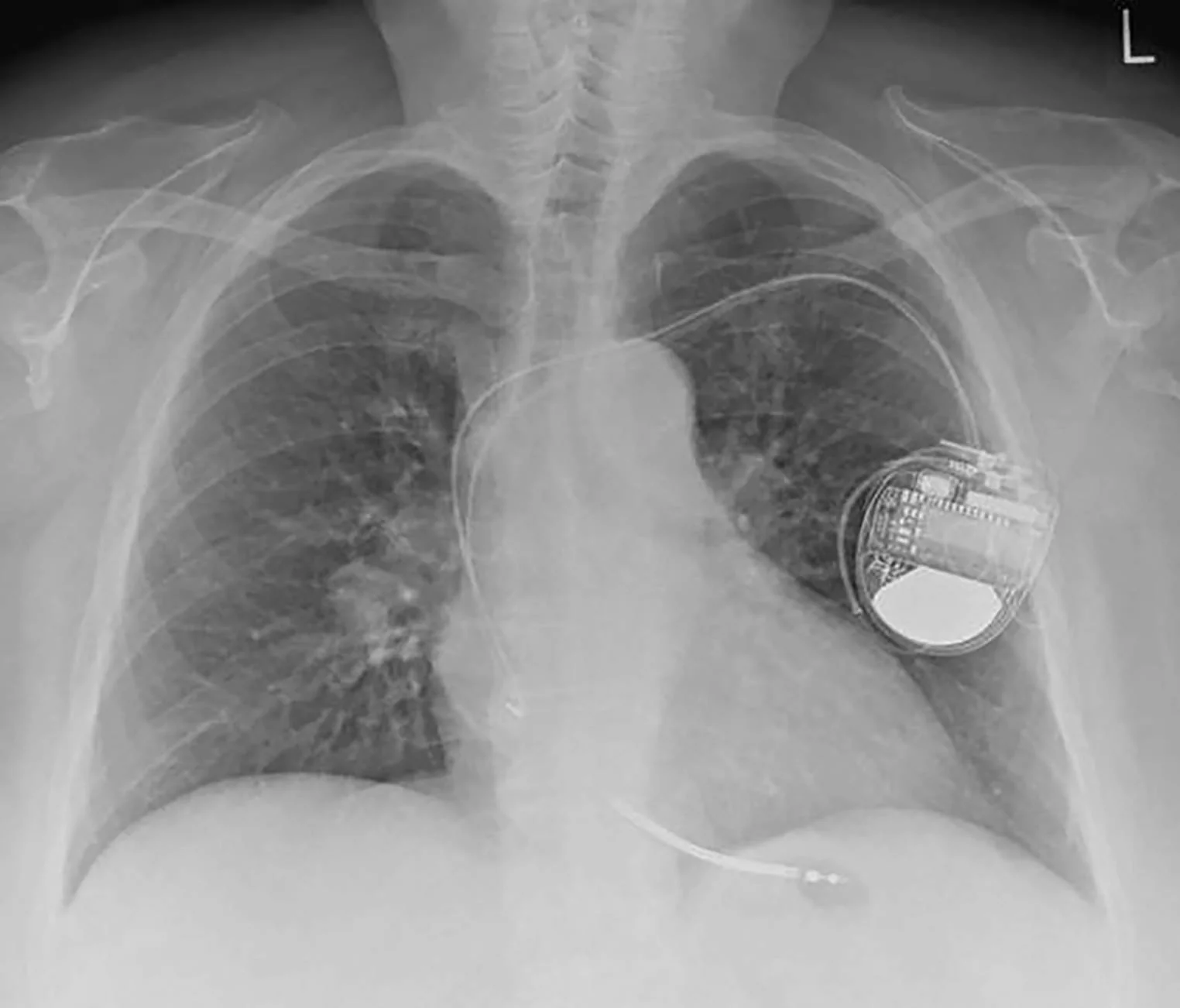
Thoraxröntgenaufnahme mit Projektion eines transvenösen ICD von links pektoral mit 2 Elektroden in Projektion auf den rechten Vorhof und rechten Ventrikel [7]

Analog zu den Herzschrittmachern kommen in der klinischen Praxis überwiegend konventionelle, transvenöse ICD (TV-ICD) zum Einsatz (Abb. 2). Diese machen nach wie vor rund 90 % aller ICD-Implantationen aus. Gründe hierfür sind die langjährige klinische Erfahrung, die sehr hohe Zuverlässigkeit der Diagnostik und Therapie sowie die breite Palette an Programmiermöglichkeiten [15, 16, 17].

## Subkutane und extravaskuläre ICD

**Abb. 3:**
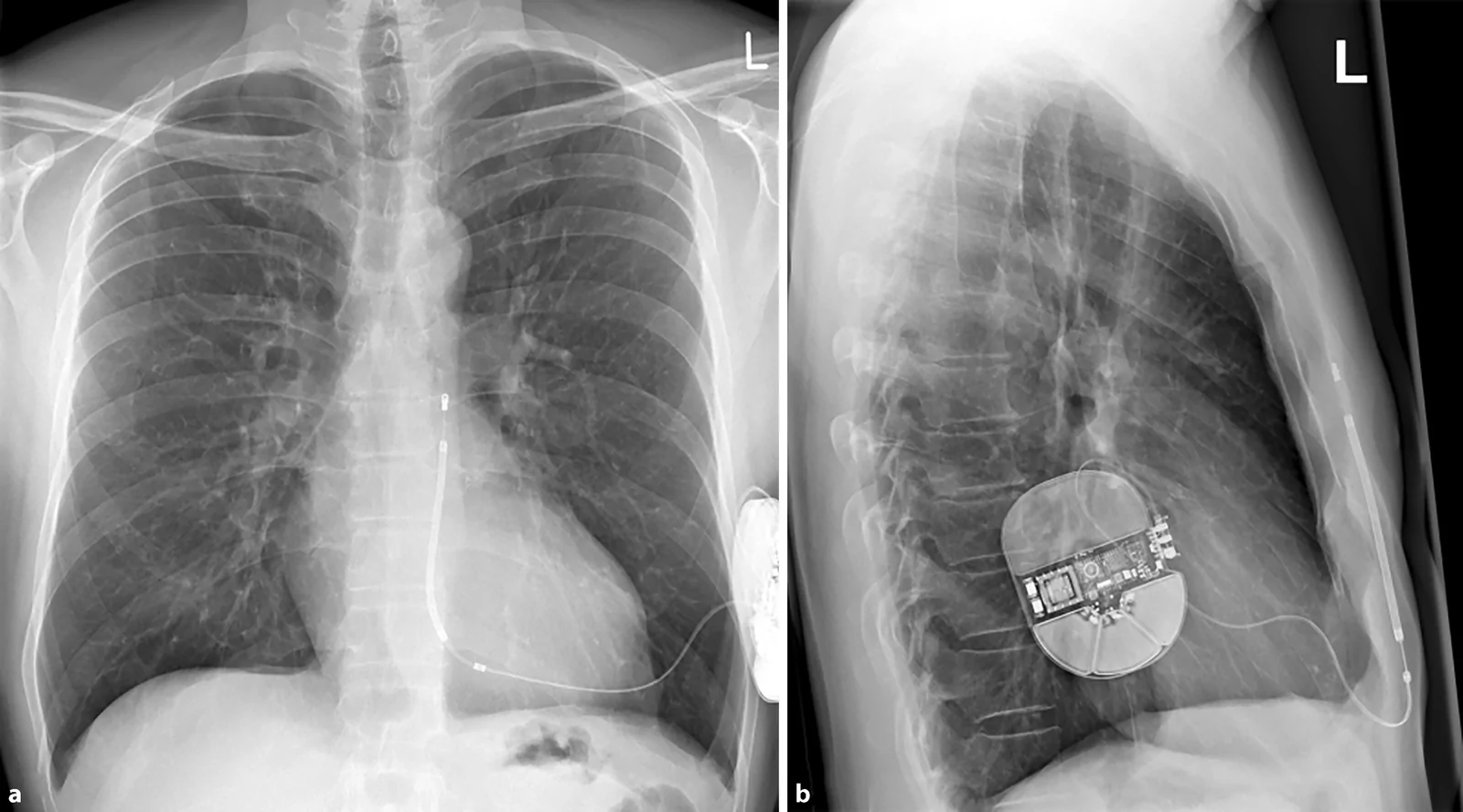
Thoraxröntgenaufnahme mit Projektion eines subkutanen implantierten Kardioverter-Defibrillators (ICD) **a** p.-a.-Röntgenaufnahme, **b** seitliche Röntgenaufnahme [7]

Im Jahr 2009 wurde in Großbritannien erstmals ein subkutaner ICD (S-ICD) implantiert. Diese Gerätevariante stellt insbesondere bei jungen Patienten oder nach Geräte-Endokarditiden sowie komplexen venösen Verhältnissen eine Alternative dar. Das Aggregat wird dabei lateral in der linken Thoraxwand subkutan bzw. intermuskulär implantiert. Von dort aus verläuft eine subkutane Elektrode unterhalb der Brust in parasternaler Lage, über die sowohl die Signalwahrnehmung als auch die Schockabgabe erfolgen (Abb. 3). Aufgrund der spezifischen Sondenposition bestehen jedoch funktionelle Limitationen. So sind weder eine Stimulation bei Bradykardie noch **antitachykardes Pacing**

antitachykardes Pacing

(ATP) möglich. Darüber hinaus ist die korrekte Detektion von Kammerflimmern bei gleichzeitigem Vermeiden von inadäquaten ICD-Schocks technisch tendenziell schwieriger im Vergleich zu transvenösen ICD-Systemen, dies aufgrund der größeren Distanz zum Myokard und endogenen wie exogenen elektrischen Störquellen durch Muskelaktivität bzw. elektromagnetische Interferenzen (EMI) durch elektronische Geräte [18].

Seit 2023 stehen zudem sogenannte extravaskuläre ICD-Systeme (EV-ICD) zur Verfügung, die über die laterale Thoraxwand – meist ebenfalls linksseitig – substernal implantiert werden. Im Vergleich zum S‑ICD ermöglicht diese Platzierung eine präzisere Signalwahrnehmung, ist jedoch mit potenziell schwerwiegenderen Komplikationen im Rahmen der Implantation assoziiert, darunter Perikard- oder Pleuraperforationen sowie Blutungen, und dem Risiko einer **Mediastinitis**

Mediastinitis.

Vorteilhaft dagegen ist bei diesen Geräten die Möglichkeit zum Pacing bei Bradykardie sowie zum ATP [19].

### Merke

Subkutane und extravaskuläre ICD sind eine potenzielle Alternative zu transvenösen Systemen v. a. bei jungen und/oder infektgefährdeten Patienten, müssen jedoch ggf. auf Bradykardie-Pacing und ATP verzichten.

### Infobox 2 Nerd-Wissen.

*Antitachykardes Pacing (ATP)*

Wenn eine *monomorphe ventrikuläre Tachykardie (VT)* detektiert wird, gibt das Gerät *kurze elektrische Impulse* ab. Deren Frequenz ist schneller, als die Frequenz der VT. Dadurch soll der Reentry-Mechanismus unterbrochen werden, ohne einen Defibrillationsschock abzugeben. Damit ist es für den Patienten schmerzfrei und bedarf für das Gerät eines viel geringeren Energieverbrauchs als eine Schockabgabe. Dieses Prinzip funktioniert nur, wenn das Gerät *präzise pacing-fähig ist* – typischerweise über eine *intrakardiale Elektrode im rechten Ventrikel* – etwas, das der S‑ICD eben nicht bietet.

## „Loop Recorder“

Ein **Ereignisrecorder**

Ereignisrecorder,

im Englischen als Loop Recorder bezeichnet, besteht aus einem subkutanen Implantat, das in der Herzregion platziert wird. Mit einer Batterielaufzeit von ca. 3 bis 4 Jahren und einer bedingten MRT-Kompatibilität erfasst das Gerät kontinuierlich relevante Rhythmusstörungen wie beispielsweise längere Pausen, Brady- oder Tachykardien. Die Daten können entweder drahtlos in der kardiologischen Praxis oder telemedizinisch ausgelesen werden.

Vor elektiven Operationen sollte (v. a. bei fehlender telemedizinischer Anbindung) der Loop Recorder ausgelesen werden, da die Signale während chirurgischer Eingriffe unter Einsatz elektrochirurgischer Geräte fehlinterpretiert oder bereits gespeicherte Daten überschrieben werden könnten. Abgesehen davon sind perioperativ keine besonderen Maßnahmen nötig [20].

## Intraoperative Komplikationen mit CIED

### Elektromagnetische Interferenzen

**Elektromagnetische Interferenzen**

**Abb. 4:**
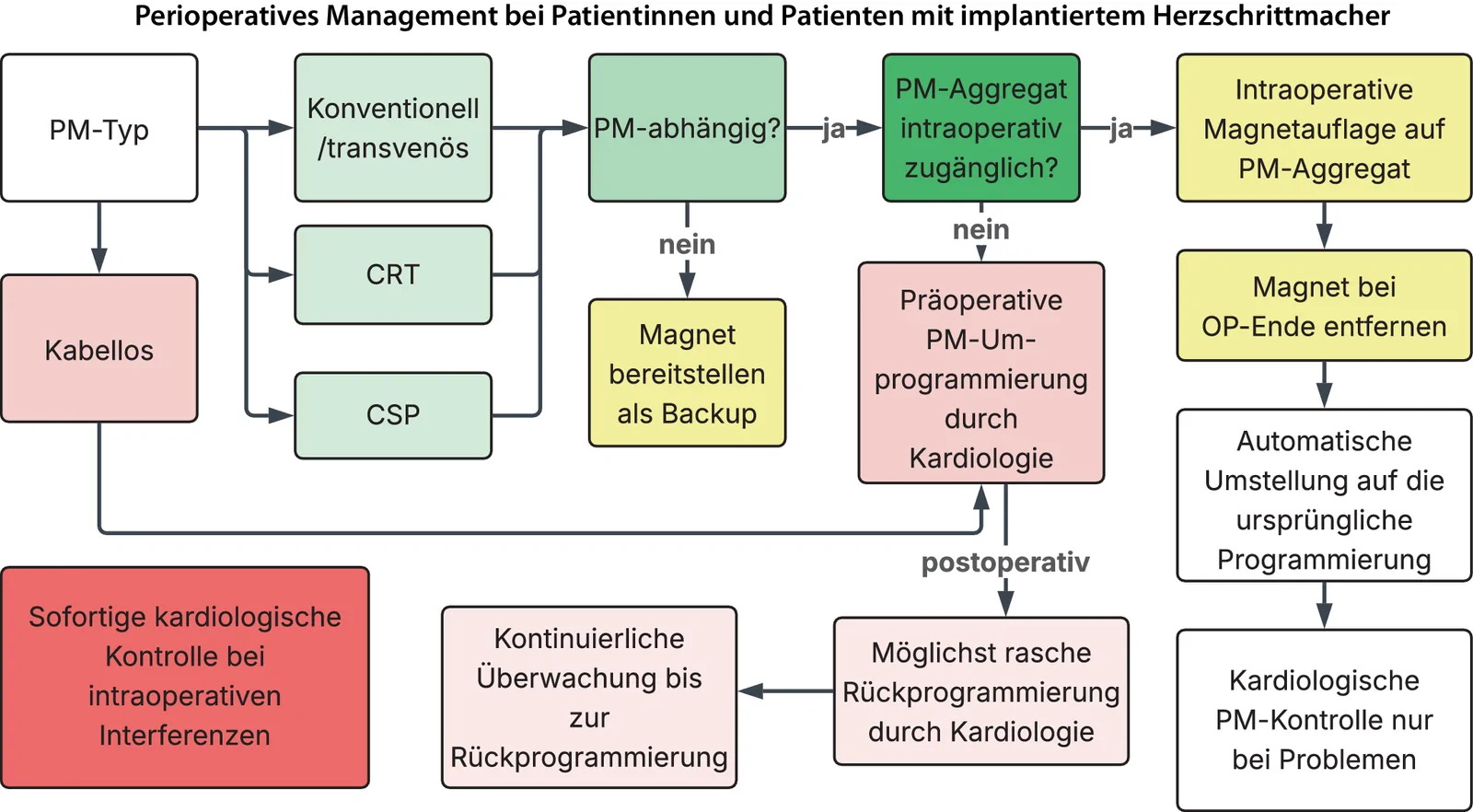
Flowchart zum perioperativen Management bei Patientinnen und Patienten mit implantiertem Herzschrittmacher. *PM* Schrittmacher, *CRT* Gerät zur kardialen Resynchronisationstherapie, *CSP* Gerät zum Conduction System Pacing. Die Abbildung ist als downloadbares PDF an der online Version des Artikels verfügbar

**Abb. 5:**
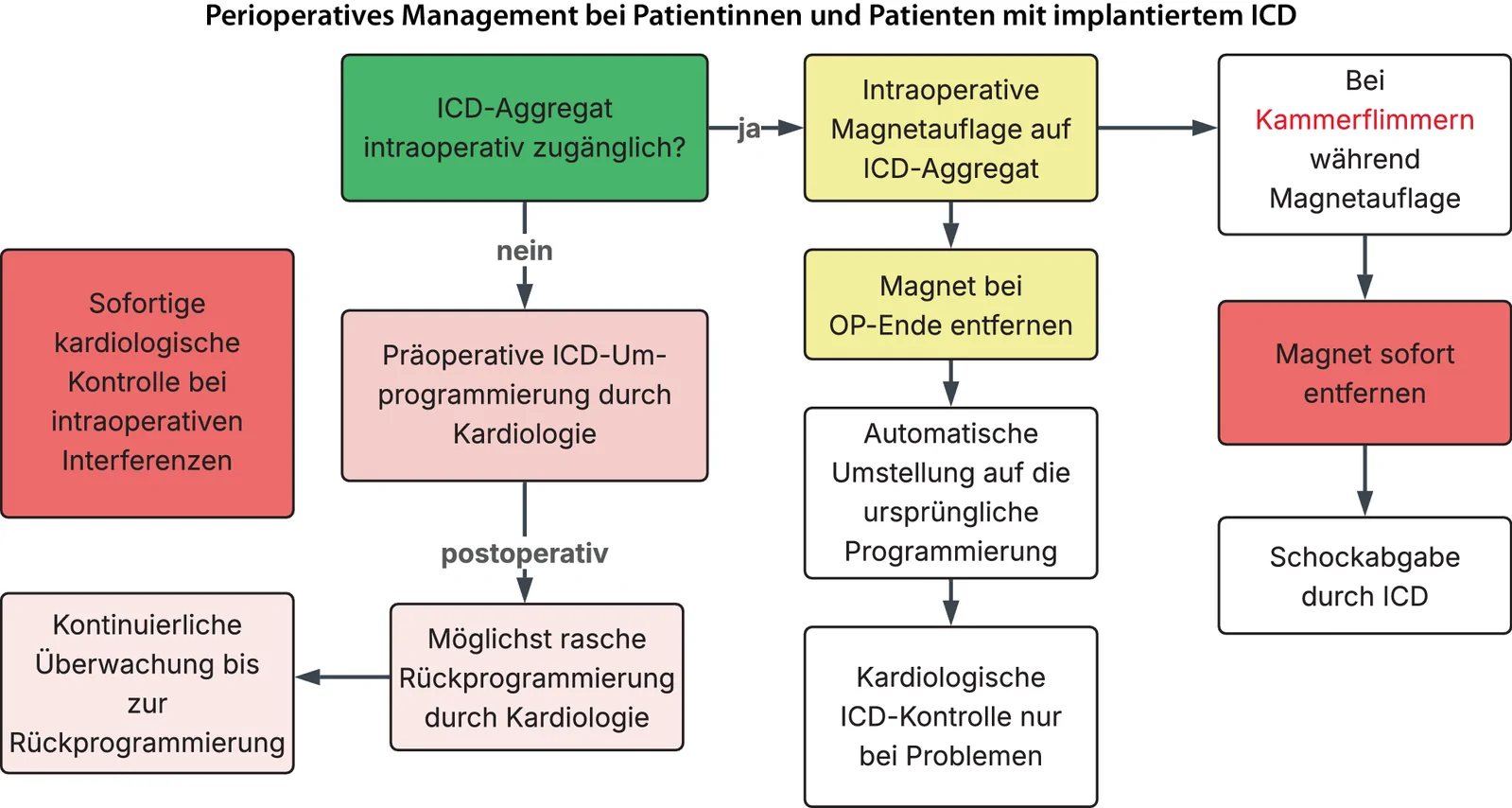
Flowchart zum perioperativen Management bei Patientinnen und Patienten mit implantiertem Kardioverter-Defibrillator (ICD). Die Abbildung ist als downloadbares PDF an der online Version des Artikels verfügbar

Elektromagnetische Interferenzen

(EMI), insbesondere durch den Einsatz monopolarer Elektrokautergeräte, können die Funktionen der CIED intraoperativ erheblich beeinflussen. Bei Schrittmachern kann es dabei zu Überwahrnehmung der Kauterströme kommen, die dann potenziell notwendige Stimulationsimpulse unterdrücken. Bei ICD können die Impulse eines Elektrokauters fälschlicherweise als Kammerflimmern fehlinterpretiert werden, was eine inadäquate Schockabgabe zur Folge haben kann, sogar mit evtl. resultierender Induktion von Kammerflimmern. Das Risiko für EMI steigt mit abnehmendem Abstand zwischen Elektrokauter und Aggregat sowie der Verwendung monopolaren Stroms. Zur Prävention von EMI muss eine präoperative Umprogrammierung oder intraoperative Magnetauflage stattfinden. Die Entscheidungsfindung anhand einer präoperativen Risikoevaluation ist im Flowchart *Perioperatives Management bei Herzschrittmachern bzw. ICD* (Abb. 4, 5) dargestellt. Die Neutralelektrode für monopolare Elektrochirurgie sollte dabei möglichst weit vom CIED entfernt platziert werden. Ein Mindestabstand von 15 cm wird in den Leitlinien empfohlen, wobei sich die Distanzangaben jeweils auf das Aggregat beziehen [21].

### Oversensing

Unter **Oversensing**

Oversensing

versteht man die fehlerhafte Wahrnehmung von extra- oder intrakardialen Signalen als Vorhof- oder Ventrikelaktivität, was zu inadäquater Therapieabgabe (im Falle eines ICD) oder -unterdrückung führen kann. Wenn Signale fälschlicherweise als Herzaktionen interpretiert werden und die Stimulation ausbleibt, kann dies bei schrittmacherabhängigen Patientinnen und Patienten instantan zu Bradykardie oder Asystolie führen. Ebenso kann Oversensing zu Tachykardien führen, beispielsweise wenn ein DDD-Schrittmacher elektrische Aktivität im Vorhof fehlerhaft erkennt und daraufhin den Ventrikel inadäquat tachykard stimuliert. Diese Komplikation tritt jedoch aufgrund eines eingebauten Noise-Reversion-Modus inzwischen äußerst selten auf. Bei Trägerinnen und Trägern einer CRT kann Oversensing zu asynchroner Stimulation und in der Folge zu akuter kardialer Dekompensation führen [12].

### Undersensing

**Undersensing**

Undersensing

bezeichnet die unzureichende Wahrnehmung intrakardialer elektrischer Signale durch ein CIED, obwohl diese im Myokard auftreten. Bei Schrittmacherträgerinnen und -trägern resultiert dies in inadäquatem Pacing, da der vorhandene Rhythmus fälschlich als Bradykardie oder Asystolie interpretiert wird. Bei ICD-Patientinnen und -Patienten kann beispielsweise ein Kammerflimmern nicht erkannt werden und damit eine Schockabgabe unterbleiben [22]. Aus anästhesiologischer Sicht ist zudem noch besonders zu beachten, dass die Signalwahrnehmung aufgrund von Einflüssen auf die intrinsische Signalamplitude z. B. durch Hyperkapnie, Hypernatriämie, Hypokaliämie, Hypoxie oder die Gabe von Mineralokortikoiden erschwert werden kann und es dadurch zu einem Undersensing kommt [23].

### Impedanzerhöhung

Verschiedene Einflussfaktoren, wie beispielsweise durch EMI ausgelöste lokale Hitzeentwicklung im Bereich der Elektroden, können zu Ödembildung und Impedanzerhöhung zwischen Elektrode und Myokard führen. In der Folge kann es zu einem sog. **Exit Block**

**Abb. 6:**
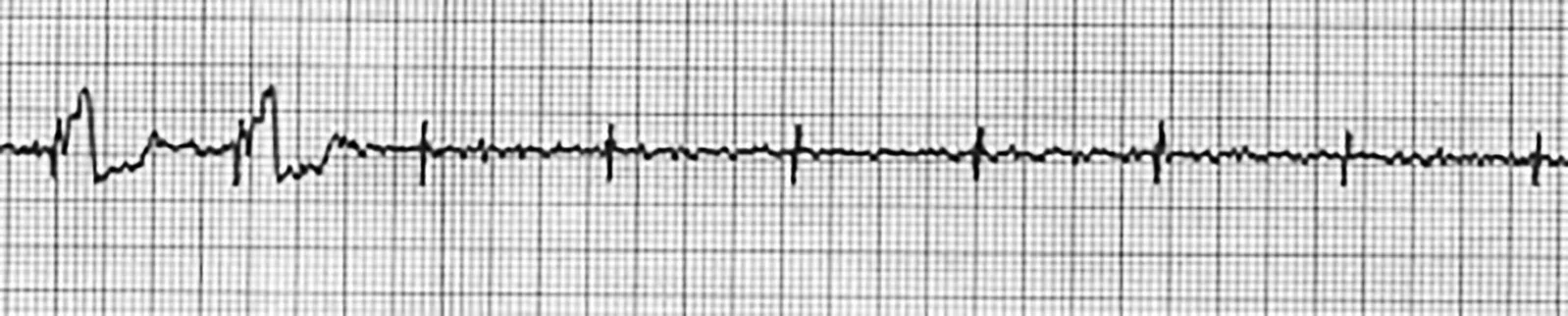
Exit block ab dem 3. Schrittmacher-Stimulationsimpuls. Auf den Schrittmacherimpuls folgt keine myokardiale Reizantwort

Exit Block

kommen. Dabei steigt die Reizschwelle derart an, dass auf abgegebene Stimulationsimpulse keine adäquate Reizantwort mehr erfolgt (Abb. 6; [12]).

#### Merke

EMI können bei CIED u. a. durch Oversensing oder asynchrone Stimulation zu potenziell lebensbedrohlichen Konsequenzen führen.

## Management von Komplikationen

Ein etabliertes Verfahren zur Prävention perioperativer Komplikationen ist die Applikation eines medizinischen Magneten auf das Aggregat. **Transvenöse Schrittmachersysteme**

Transvenöse Schrittmachersysteme

lassen sich hierdurch in der Regel in einen asynchronen Stimulationsmodus (V00/A00) überführen, wodurch Störungen durch Artefakte im Sinne eines Oversensing vermieden werden. Nach Entfernung des Magneten kehrt das Gerät in den zuvor programmierten Betriebsmodus zurück. Bei Magnetauflage ist aber zu berücksichtigen, dass ein Schrittmacher im asynchronen Stimulationsmodus – sei es durch gezielte Umprogrammierung oder durch die Applikation eines Magneten – ein potenzielles proarrhythmogenes Risiko birgt. Insbesondere bei erhöhter intrinsischer Herzaktivität und daraus resultierender Tachykardie (z. B. während Narkoseein- oder Narkoseausleitung oder bei Hypovolämie) kann es zu inadäquaten Fusionen oder Überlagerungen durch die Schrittmacherstimulation kommen.

### Cave

Vorsicht! Magnetauflage bei Schrittmachern: Herzschrittmacher enthalten einen magnetfeldempfindlichen Sensor. Wird ein statisches Magnetfeld ≥ 10 Gauß in unmittelbarer Nähe erzeugt (z. B. durch einen medizinischen Magneten), aktiviert der Schrittmacher einen vorprogrammierten Modus, welcher meist aus einer fixen Stimulationsfrequenz ohne Wahrnehmung der kardialen Eigenaktivität besteht. Dadurch werden EMI vermieden, durch die Asynchronität besteht allerdings das Risiko der Auslösung von Arrhythmien.

Wenige Schrittmachermodelle (v. a. solche des Herstellers Biotronik) reagieren jedoch nicht mit einem asynchronen Stimulationsmodus auf eine Magnetauflage [24]. Angaben zu diesem sehr seltenen Fall sollten bei Bedarf bei der behandelnden Kardiologin oder dem behandelnden Kardiologen erfragt und im Protokoll der präoperativen Abklärung gut erkennbar dokumentiert werden.

Auch bei **kabellosen Schrittmachern**

kabellosen Schrittmachern

ist eine Umschaltung in den asynchronen Stimulationsmodus durch Magnetapplikation bei gewissen Herstellermarken (Medtronic) nicht möglich. Der Umgang mit diesen Systemen erfordert daher vor Eingriffen mit potenzieller EMI eine gezielte Umprogrammierung in einen asynchronen Stimulationsmodus durch geschultes Fachpersonal. Nur bei gesicherter Kenntnis des exakten Gerätemodells und eindeutigen Herstellerangaben kann im Ausnahmefall auf eine Umprogrammierung verzichtet werden.

Bei den **CRT‑D**

CRT‑D

führt die Magnetauflage ebenfalls nicht automatisch zu einem asynchronen Stimulationsmodus. Schrittmacherabhängige Patientinnen und Patienten sind daher trotz Magnetapplikation nicht vor EMI-bedingter Stimulationsinhibition geschützt. Eine präoperative Umprogrammierung in den asynchronen Stimulationsmodus ist daher bei Patienten mit CRT‑D – besonders wenn sie Schrittmacher-abhängig sind – obligat. Im Gegensatz dazu verhalten sich **CRT‑P**

CRT‑P

auch bei Magnetauflage wie andere Schrittmacher.

Die Magnetantwort bei **CSP-Systemen**

CSP-Systemen

entspricht jener von transvenösen Schrittmachern und sie stimulieren bei Magnetauflage in einem asynchronen Modus.

Bei **ICD**

ICD

bewirkt die Magnetapplikation eine Deaktivierung sämtlicher Antitachykardie-Funktionen. Arrhythmien werden zwar weiterhin erkannt, jedoch nicht therapiert. Kommt es bei aufliegendem Magneten zu Kammerflimmern, muss daher der Magnet unverzüglich entfernt werden, um eine adäquate Schockabgabe durch den ICD zu ermöglichen. Falls dies nicht gelingt, muss extern defibrilliert werden.

Implantierbare **Loop Recorder**

Loop Recorder

besitzen ausschließlich eine Dokumentationsfunktion, sodass perioperativ keine relevanten Komplikationen zu erwarten sind.

Bei notfallmäßigen operativen Eingriffen ist das potenzielle Risiko einer EMI-bedingten CIED-Fehlfunktion stets gegen die unmittelbare vital bedrohliche Indikation zur Operation oder Intervention ohne den Zeitverlust Umprogrammierung abzuwägen. In jedem Fall muss ein medizinischer Magnet im OP oder im Interventionsraum bereitgehalten werden.

Tab. 2 bietet eine Übersicht über die Notfallmaßnahmen bei intraoperativen Komplikationen.

**Tab. 2:** Notfallmaßnahmen bei intraoperativen Komplikationen.

Komplikation	Maßnahmen
*Oversensing:* Bradykardie, Asystolie, inadäquate Schockabgaben; bei CRT-Geräten: asynchrone Stimulation mit kardialer Dekompensation	EMI-Quelle entfernen (Chirurgische Stromanwendung stoppen)
	Magnetauflage
	Externes Pacing
	Externe Defibrillation (wenn möglich, Pads > 15 cm vom CIED entfernt)
*Undersensing:* Schrittmacher: inadäquates Pacing ICD: fehlende Erkennung von Kammerflimmern	EMI-Quelle entfernen (Chirurgische Stromanwendung stoppen)
	Magnetauflage
	Externes Pacing
	Externe Defibrillation (Wenn möglich, Pads > 15 cm vom CIED entfernt)
	Korrektur reversibler Faktoren (Oxygenierung, Elektrolyte, Säure-Basen-Status)
*Impedanzerhöhung, Exit Block, Aggregaterschöpfung:* Eingeschränkte Impulsweiterleitung, Verlust der Stimulation	Externes Pacing
	Externe Defibrillation (Wenn möglich, Pads > 15 cm vom CIED entfernt)
*Maligne Herzrhythmusstörung bei Schrittmachergeräten:* Kammerflimmern, Torsade de Pointes, höhergradige AV-Blockierungen	Magnetentfernung
	Externe Defibrillation (Wenn möglich, Pads > 15 cm vom CIED entfernt)
*Fehlende Erkennung von Kammerflimmern bei ICD*	EMI-Quelle entfernen (Chirurgische Stromanwendung stoppen)
	Magnetentfernung, falls nicht erfolgreich externe Defibrillation (Wenn möglich, Pads > 15 cm vom CIED entfernt)
	Korrektur reversibler Faktoren (Oxygenierung, Elektrolyte, Säure-Basen-Status)

*CIED* kardial implantierbare elektronische Geräte, *CRT* kardiale Resynchronisationstherapie, *EMI* elektromagnetische Interferenz, *ICD* implantierbarer Kardioverter-Defibrillator

### Merke

Kabellose Schrittmacher müssen vor Eingriffen mit dem Risiko für elektromagnetische Interferenzen häufig umprogrammiert werden. CRT‑P und CSP können wie alle anderen Schrittmacher gehandhabt werden, CRT‑D wie ICD.

### Fallbeispiel Fortsetzung

Am Operationstag erfolgt die komplikationslose Einleitung einer Vollnarkose. Ein Magnet wird am Anästhesie-Arbeitsplatz bereitgehalten. Jedoch wird vom Anästhesie-Team vergessen, das Operationsteam über den vorhandenen Schrittmacher und die nötigen Maßnahmen zu informieren, sodass die Neutralelektrode für monopolare Elektrochirurgie an der linken Schulter montiert wird. Bereits während des Hautschnittes mit dem Elektrokauter kommt es plötzlich zu einem kombinierten Blutdruck- und etCO_2_-Abfall. Das EKG zeigt eine von Artefakten durchzogene Kurve, die Pulswellen eine Bradykardie von 27/min. Das aufmerksame Anästhesieteam erkennt die elektromagnetische Interferenz und reagiert sofort. Es informiert das chirurgische Team, welches die Operation kurzzeitig pausiert, und legt den Magneten auf das Schrittmacheraggregat. Blutdruck und etCO_2_ steigen rasch wieder an, EKG und Pulskurve zeigen eine regelmäßige Herzfrequenz von 80/min an. Die weitere Operation verläuft problemlos bei aufliegendem Magneten. Als sich der Patient postoperativ im Aufwachraum befindet, erfolgt eine kardiologische Visite zur Funktionskontrolle des Schrittmachers.

## Narkoseverfahren und Medikamente

Alle üblichen anästhesiologischen Verfahren sind bei CIED-Patientinnen und -Patienten grundsätzlich sicher einsetzbar. Bei jeglichen frisch implantierten Geräten kann Lachgas eine Dilatation der subkutanen Tasche verursachen – mit (theoretisch) möglicher Gerätedislokation und Kontaktverlust – und soll daher vermieden werden [25]. Neutralelektroden von Nervenstimulatoren sollten möglichst fern vom CIED platziert und Stromflüsse vom Device weggeleitet werden. Alle Lokalanästhetika sowie bestimmte Antiarrhythmika (Propafenon, Flecainid) erhöhen die Reizschwelle des Myokards, was u. U. zu einer fehlenden Erregung durch die Schrittmacherstimulation führen kann. Darüber hinaus können Sympathomimetika die Entstehung von Tachyarrhythmien begünstigen. Faszikulationen nach Gabe von Succinylcholin, Myoklonien durch Verwendung von Etomidat und postoperatives Shivering können ein Oversensing und daraus resultierende inadäquate Schockabgaben verursachen [23, 26].

Aufgrund der bereits beschriebenen potenziellen Komplikationen ist es bei CIED-Trägerinnen und -Trägern erforderlich, perioperativ jederzeit einen medizinischen Magneten verfügbar zu halten bzw. diesen proaktiv zu nutzen.

## Thorakale Interventionen und Operationen

Thoraxchirurgische Eingriffe stellen aufgrund der anatomischen Nähe zum CIED eine besondere Herausforderung dar. Das kontinuierliche Monitoring von Hämodynamik und Herzrhythmus (Blutdruckmessung, EKG, Sauerstoffsättigung) ist obligat. Die Gefahr einer postoperativen Infektion, die das Aggregat oder die Sonden betrifft, ist im Vergleich zu anderen Operationen erhöht. Umfangreiche Resektionen, Tumoroperationen oder Herzoperationen können zu Dislokationen der CIED-Bestandteile führen und eine Revision oder Explantation erforderlich machen [27]. Magnettücher zur Ablage chirurgischer Instrumente dürfen keinesfalls über dem Thorax positioniert werden [28].

## Zentralvenöse Zugänge

Die Indikation zur Anlage zentralvenöser Zugänge sollte bei Patientinnen und Patienten mit CIED restriktiv gestellt werden. Soweit notwendig, ist eine kontralaterale Platzierung gegenüber dem CIED zu bevorzugen. Einführen oder Entfernen von Pulmonaliskathetern kann zur Dislokation insbesondere von frisch implantierten Sonden führen, weshalb Manipulationen radiologisch kontrolliert erfolgen sollten. Sämtliche zentralvenöse Zugänge müssen unter strikt aseptischen Bedingungen gelegt werden, um Bakteriämien und mögliche konsekutive Sondeninfektionen zu vermeiden [26].

### Merke

Bei CIED-Patientinnen und -Patienten sind alle üblichen Anästhesieverfahren sicher anwendbar, erfordern jedoch besondere Vorsicht bei frisch implantierten Geräten, thorakalen Eingriffen und zentralvenösen Zugängen sowie konsequentes Monitoring und Verfügbarkeit eines Magneten.

## Spezielle Situationen

### Bildgebung

Vor einer Magnetresonanztomographie(MRT)-Untersuchung bei Patientinnen und Patienten mit CIED muss zunächst die MRT-Kompatibilität des Geräts überprüft werden. Die häufig befürchteten direkten mechanischen Auswirkungen auf ein CIED sind in der Regel vernachlässigbar. Die mechanische Belastung durch das Magnetfeld eines 1,5-Tesla-MRT entspricht in etwa der Krafteinwirkung wie durch die Schwerkraft oder Beschleunigungskräfte z. B. beim Autofahren [29]. Dabei gilt es zu beachten, dass Interferenzen in jeder MRT-Untersuchung grundsätzlich auftreten können, jedoch insbesondere bei Patientinnen und Patienten, die auf einen Schrittmacher angewiesen sind, zu relevanten Komplikationen führen können [30].

Die MRT-Kompatibilität eines CIED wird durch den Hersteller definiert und ist üblicherweise im Gerätepass oder im ärztlichen Bericht vermerkt. Es sei darauf hingewiesen, dass auch Patienten mit formal nicht als MRT-kompatibel gekennzeichneten Geräten in der Praxis häufig ohne Probleme einer MRT-Untersuchung unterzogen werden können, vorausgesetzt, das Untersuchungszentrum verfügt über die entsprechenden etablierten Protokolle und Expertise. MRT-Untersuchungen sind dagegen grundsätzlich kontraindiziert bei chirurgisch-epikardialen Sonden sowie bei verbliebenen, veralteten oder defekten Sonden. In Ausnahmefällen kann eine MRT-Untersuchung jedoch sogar in diesen Situationen durchgeführt werden, sofern das Nutzen-Risiko-Verhältnis günstig ist. Eine solche Entscheidung sollte stets durch eine auf CIED spezialisierte Kardiologin oder einen Kardiologen getroffen werden [30]. Eine Umprogrammierung der CIED (zur Vermeidung von Oversensing oder inadäquaten Schocks) ist meist erforderlich, kontinuierliche EKG-Überwachung während der Untersuchung sowie eine nachfolgende Gerätekontrolle werden empfohlen. Eine aktuelle Übersicht zur MRT-Tauglichkeit ist unter www.mrisafety.com verfügbar [31].

Für Sonographie, Röntgen und CT stellen CIED grundsätzlich kein Hindernis dar. Einzelne Fallberichte beschreiben EMI bei außergewöhnlich hoher Strahlendosis während CT-Untersuchungen; aufgrund der sehr niedrigen Inzidenz gilt das Risiko allerdings als äußerst gering. Artefakte in den Bildaufnahmen sind jedoch möglich [32].

### Akuter Myokardinfarkt bei implantiertem Schrittmacher

Ein CIED kann die Diagnostik eines akuten Myokardinfarkts erschweren, da typische EKG-Veränderungen u. U. während ventrikulärer Stimulation verschleiert werden können. Das 12-Kanal-EKG bleibt jedoch Standard und sollte innerhalb der ersten 10 min nach Aufnahme erfolgen. Dessen Bewertung erfolgt anhand der Sgarbossa-Kriterien, welche auch bei CIED-Trägerinnen und -Trägern hochspezifisch sind [33].

### Defibrillation und Kardioversion

CIED tolerieren in der Regel eine externe Defibrillation oder Kardioversion des Patienten. Schäden sind selten, jedoch steigt das Risiko mit der Energiehöhe sowie der Nähe der Defibrillator-Pads zum CIED-Aggregat. Eine Erhöhung der Stimulationsschwelle oder Myokardschäden sind ebenfalls möglich. Biphasische Defibrillation wird bevorzugt. Außerdem ist ein Mindestabstand von 15 cm zwischen CIED und Defibrillator-Pads einzuhalten. Die Positionierung der Defibrillator-Elektroden sollte den Stromfluss am Aggregat vorbeileiten.

#### Merke

Bei notfallmäßigen Fragen kann meist die diensthabende Person der Rhythmologie des nächstgelegenen Zentrumsspitals weiterhelfen.

## Übersicht zum perioperativen anästhesiologischen Management von Patientinnen und Patienten mit CIED

1. *Sichtung* *der Vorerkrankungen der Patientin bzw. des Patienten inkl. Indikation für das CIED*
2. *Device-Identifikation*
  Dokumentation oder Ausweis kontrollieren (Hersteller, Typ (*PM oder ICD*), Funktion)
  Wenn unklar: Thoraxröntgen (zur genauen Lokalisierung von Aggregat und Sonden) + Nachfrage bei Kardiologie
3. *Zeitpunkt der letzten Funktionsabfrage:*
  > 6 Monate zurück: Abfrage durch Device-Techniker oder Kardiologie: Batterie, Modi, Sensing, Reizschwellen, Schrittmacherabhängigkeit
  < 6 Monate: keine Abfrage notwendig
4. *Ggf. Durchführung eines 12-Kanal-EKG mit Rhythmusstreifen*
5. *Kontrolle der Serum-Elektrolyt-Werte sowie ggf. des Säure-Basen Status*
6. *EMI-Risiko einschätzen*
  Elektrochirurgie, MRT etc.
  Abstand des Operationsgebietes und der Neutralelektrode zum CIED (> 15 cm empfohlen)
  Hohe Vorsicht bei ICD/CRT-D: Therapieauslösung durch Artefakte möglich
  EMI-Risiko bei allen Patientinnen und Patienten gleich, jedoch gefährlichere Konsequenzen bei schrittmacherabhängigen Personen
7. *Präoperative Umprogrammierung des CIED, bei*
  kabellosen Schrittmachern ohne Magnetmodus
  ICD und Elektrochirurgie, wenn keine Möglichkeit zur Magnetauflage besteht bzw. die ICD-Patientin oder der ICD-Patient schrittmacherabhängig ist (zwingende Umprogrammierung!)
  Operationen in Bauchlage und anderweitiger Unzugänglichkeit des CIED
8. *Magnetmanagement planen*
  Bei transvenösen Schrittmachern *ohne* Schrittmacherabhängigkeit: Magnet bereithalten, nur auflegen bei Verdacht auf EMI
  Bei transvenösen Schrittmachern *mit* Schrittmacherabhängigkeit: Magnet auflegen
  Bei ICD (*ohne* Schrittmacherabhängigkeit) ohne präoperative Umprogrammierung: Magnet auflegen
  Kenntnis, wie das Gerät auf Magneten reagiert (z. B. bei ICD: Deaktivierung der Schockfunktion)
  Achtung: Der Magnet deaktiviert *nicht* das Pacing!
9. *Intraoperative Strategie*
  Kontinuierliches EKG- und S_p_O_2_-Monitoring
  Elektrochirurgie möglichst bipolar oder Verwendung eines Ultraschallskalpells
  Bei monopolarer Elektrochirurgie Neutralelektrode fern vom Device (> 15 cm) platzieren, Stromfluss vom Device wegführend
  Defibrillator/Externe Pacing-Bereitschaft sicherstellen
  Prophylaktisches Aufkleben von Defibrillator-Pads in Hochrisikosituationen (Schrittmacher-abhängige Patientinnen und Patienten bei CIED-naher Chirurgie (< 15 cm Abstand) oder Unzugänglichkeit des Gerätes sowie Risiko einer Impedanzerhöhung mit Exit-Block)
  Kommunikation mit OP-Team im präoperativen Team-Time-Out über das Vorgehen bei intraoperativen CIED-Komplikationen
10. *Postoperative Kontrolle*
  Sofortige oder zumindest zeitnahe Nachkontrolle durch Kardiologie bei präoperativer Umprogrammierung. Kontinuierliche Überwachung am Monitor bis zur Kontrolle.
  Device-Kontrolle gemäß hausinternen Richtlinien.
  Unmittelbare oder zeitnah postoperative Kontrolle nach offensichtlichem Auftreten von EMI. Kontinuierliche Überwachung am Monitor sowie externe Pacing‑/Defibrillationsbereitschaft bis zur Kontrolle.

### Merke

Im Zweifelsfall soll vor jedem Eingriff bei Patientinnen und Patienten mit CIED Rücksprache mit einer Fachperson der Kardiologie bzw. Rhythmologie erfolgen und ggf. eine Umprogrammierung vorgenommen werden.

## Fazit für die Praxis

- CIED-Trägerinnen und -Träger weisen häufig erhebliche kardiovaskuläre Vorerkrankungen auf, welche in der präoperativen Abklärung exakt evaluiert werden müssen.
- Von besonderer Relevanz sind die Art, Programmierung und Lage des CIED sowie die Klärung der Frage, ob der Patient Schrittmacher-abhängig ist oder nicht.
- EMI können v. a. beim Einsatz monopolarer Elektrokauter auftreten und zu Stimulationsausfall oder inadäquaten ICD-Therapien führen. Besonders bei schrittmacherabhängigen Patientinnen und Patienten ist das Risiko von Komplikationen relevant.
- Bei Patientinnen und Patienten mit transvenösen Schrittmachern sowie bei schrittmacherabhängigen Patientinnen und Patienten mit ICD wird die periprozedurale Magnetauflage empfohlen.
- Bei kabellosen Schrittmachern ist die Magnetantwort abhängig vom Gerätehersteller. Bei einem Eingriff mit einem Risiko für EMI müssen kabellose Schrittmacher häufig umprogrammiert werden. Hier empfiehlt sich die Rücksprache mit dem zuständigen kardiologischen Fachpersonal.
- Falls der ICD oder Schrittmacher intraoperativ nicht zugänglich ist (z. B. bei OP in Bauchlage oder thorakalen Eingriffen), muss eine Umprogrammierung erfolgen.
- Bei präoperativ stattgehabter Umprogrammierung jeglicher CIED soll eine kardiologische Nachkontrolle möglichst direkt postoperativ erfolgen. Bis dahin müssen eine kontinuierliche Überwachung am Monitor sowie externe Schrittmacher‑/Defibrillationsbereitschaft gewährleistet bleiben, was in der Regel einen Verbleib im Auswachraum bzw. im Intermediate-care- oder Intensivbehandlungsbereich erzwingt.

## Supplementary Information

PDF „Algorithmus zum Perioperatives Management bei ICD und Herzschrittmachern“

## CME-Fragebogen

Welcher Vorteil ist charakteristisch für kabellose Schrittmacher?

Kabellose Schrittmacher benötigen keine Batterien.

Sie haben eine unbegrenzte Lebensdauer.

Sie können jederzeit extern aufgeladen werden.

Es entfällt die Notwendigkeit transvenöser Elektroden.

Sie besitzen kein Risiko für elektromagnetische Interferenzen.

Was ist zu tun bei Erschöpfung des Generators eines kabellosen Schrittmachers?

Er schaltet automatisch in einen Energiesparmodus.

Er wird über eine subkutane Tasche ausgetauscht.

Der Generator wird entfernt und umgehend repariert.

Der Generator wird durch die Haut komplett aufgeladen.

Ein neues System wird implantiert, das alte verbleibt im Ventrikel.

Welcher Faktor erhöht das Risiko für elektromagnetische Interferenzen (EMI) bei Schrittmacher-Trägerinnen und -Trägern besonders?

Generell nur Verwendung von bipolarem Strom

Große Distanz zwischen Elektrokauter und Aggregat

Nutzung von monopolarem Strom in unmittelbarer Nähe des CIED

Kurze Nutzungsdauer der Elektrochirurgie

Magnetresonanztomographie-Sicherheit des Aggregates

Welche Hauptformen des Conduction System Pacing (CSP) gibt es?

Rechtsventrikuläres und linksventrikuläres Pacing

His-Bündel-Pacing und Linksschenkelbereichs-Stimulation

Asynchrones Pacing und synchrones Pacing

Transkutanes Pacing und transvenöses Pacing

Einkammer-Pacing und Zweikammer-Pacing

Worin besteht die Hauptgefahr elektromagnetischer Interferenzen (EMI) bei ICD-Trägern während Elektrochirurgie?

Gefährliche Überhitzung des Aggregats

Schockabgabe (fälschliche Kammerflimmer-Detektion)

Dauerhafte Signalverstärkung

Fehlende Speicherung von Ereignissen

Sofortige Batterieentladung des Device

Wie äußert sich Oversensing bei schrittmacherabhängigen Patientinnen und Patienten?

Übermäßige Signalverstärkung

Dauerstimulation beider Herzkammern

Auftreten von Bradykardie oder Asystolie

Dyssynchrone Stimulation von Vorhof und Ventrikel

Fehlende Speicherung klinisch relevanter Daten

Vor welchem bildgebenden Verfahren muss bei CIED-Trägerinnen und -Trägern meist eine erfahrene Fachperson konsultiert werden?

MRT

Röntgenbild

Sonographie

CT

Echokardiographie

Welches Risiko besteht bei thoraxchirurgischen Eingriffen bei CIED-Trägerinnen und -Trägern?

Das CIED erfüllt keine Funktion.

Die Elektroden verlieren ihre Leitfähigkeit.

Es kann ein Kurzschluss im System auftreten.

Es kann zur Dislokation oder Infektion der CIED-Komponenten kommen.

Eine vollständige Inhibition des Pacings kann vorkommen.

Wodurch kann es bei frisch implantierten CIED zu einer Dislokation kommen?

Gabe von Lachgas

Verwendung von Isofluran

Einsatz von Etomidat

Postoperatives Shivering

Hypokaliämie

Welche Maßnahme wird für das postoperative Management von Patientinnen und Patienten mit CIED empfohlen?

Routinemäßige Entfernung des Aggregates nach jeder Operation

Verzicht auf Monitorüberwachung, da postoperative EMI unwahrscheinlich sind

Prophylaktische Gabe von Antiarrhythmika zur Vermeidung von Schocks

Dauerhafte Umprogrammierung in den asynchronen Modus

Kontrolle und ggf. Auslesen des Gerätes bei auffälligem Verlauf
