# Supplementary material for: Update on the perioperative management of cardiac implantable electronic devices
Source: Anaesthesiologie. 2026 Mar 11;75(4):287–300. [Article in German] doi: 10.1007/s00101-026-01657-3 (PMC13722808; doi:10.1007/s00101-026-01657-3)
Supplement: Supplementary file 1 [file 101_2026_1657_MOESM1_ESM.pdf]

Perioperatives Management bei Kardioverter-Defibrillator

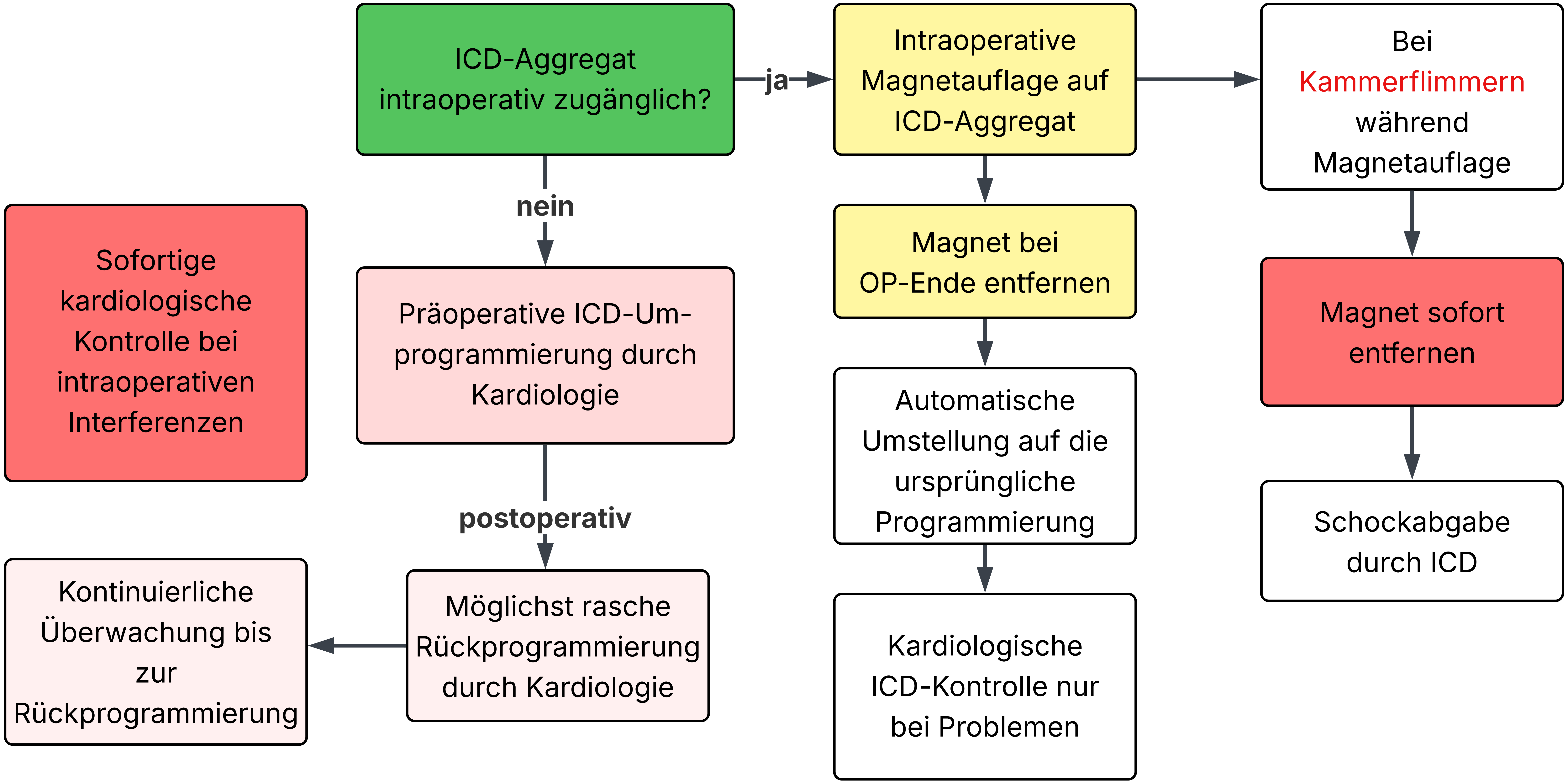

ICD: Kardioverter-Defibrillator

Zusatzmaterial zum Beitrag „**Update zum perioperativen Umgang mit kardial implantierten elektronischen Geräten**" von Fiona Uhor, Andreas Haeberlin und Jürgen Knapp (2026).
Beitrag und Zusatzmaterial stehen Ihnen auf www.springermedizin.de zur Verfügung. Bitte geben Sie dort den Beitragstitel in die Suche ein.

Die Anaesthesiologie

Perioperatives Management bei Herzschrittmachern

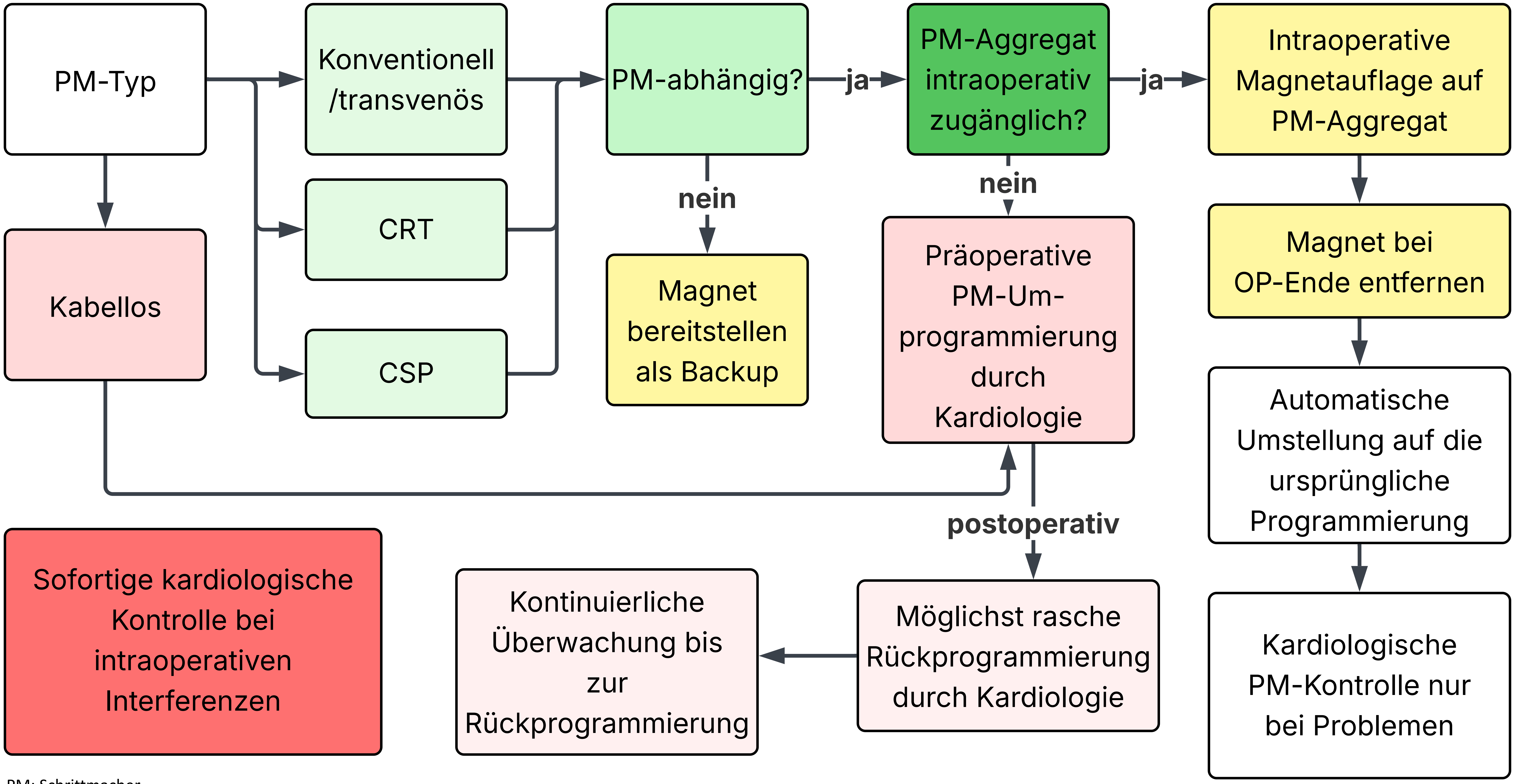

PM: Schrittmacher,
CRT: Gerät zur kardialen Resynchronisationstherapie,
CSP: Gerät zum Conduction System Pacing

Zusatzmaterial zum Beitrag **„Update zum perioperativen Umgang mit kardial implantierten elektronischen Geräten“** von Fiona Uhor, Andreas Haeberlin und Jürgen Knapp (2026).
Beitrag und Zusatzmaterial stehen Ihnen auf www.springermedizin.de zur Verfügung. Bitte geben Sie dort den Beitragstitel in die Suche ein.

Die Anaesthesiologie

Übersicht zum perioperativen anästhesiologischen Management von Patientinnen und Patienten mit CIED:

1. **Sichtung der Vorerkrankungen der Patientin bzw. des Patienten inkl. Indikation für das CIED**

2. **Device-Identifikation**
- Dokumentation oder Ausweis kontrollieren (Hersteller, Typ (PM oder ICD), Funktion)
- Wenn unklar: Röntgen-Thorax (zur genauen Lokalisierung von Aggregat und Sonden) + Nachfrage bei Kardiologie

3. **Zeitpunkt der letzten Funktionsabfrage:**
- >6 Monate zurück: Abfrage durch Device-Techniker oder Kardiologie: Batterie, Modi, Sensing, Reizschwellen, Schrittmacherabhängigkeit
- <6 Monate: keine Abfrage notwendig

4. **Ggf. Durchführung eines 12-Kanal-EKG mit Rhythmusstreifen**

5. **Kontrolle der Serum-Elektrolytwerte, sowie ggf. Säure-Basen Status**

6. **EMI-Risiko einschätzen**
- Elektrochirurgie, MRT etc.
- Abstand des Operationsgebietes und der Neutralelektrode zum CIED (>15 cm empfohlen)
- Hohe Vorsicht bei ICD/CRT-D: Therapieauslösung durch Artefakte möglich
- EMI-Risiko bei allen Patientinnen und Patienten gleich, jedoch gefährlichere Konsequenzen bei schrittmacherabhängigen Personen

7. **Präoperative Umprogrammierung des CIED, bei**
- kabellosen Schrittmachern ohne Magnetmodus
- ICD und Elektrochirurgie, wenn keine Möglichkeit zur Magnetauflage besteht bzw. die ICD-Patientin oder der ICD-Patient schrittmacherabhängig ist (zwingende Umprogrammierung!)
- Operationen in Bauchlage und anderweitiger Unzugänglichkeit des CIED

8. **Magnetmanagement planen**

- Bei transvenösen Schrittmachern ohne Schrittmacherabhängigkeit: Magnet bereithalten, nur auflegen bei Verdacht auf EMI
- Bei transvenösen Schrittmachern mit Schrittmacherabhängigkeit: Magnet auflegen
- Bei ICD (ohne Schrittmacherabhängigkeit) ohne präoperative Umprogrammierung: Magnet auflegen
- Kenntnis, wie das Gerät auf Magneten reagiert (z. B. bei ICD: Deaktivierung der Schockfunktion)
- Achtung: Der Magnet deaktiviert nicht das Pacing

9. **Intraoperative Strategie**

- Kontinuierliches EKG- und SpO_2-Monitoring
- Elektrochirurgie möglichst bipolar oder Verwendung eines Ultraschallskalpells
- Bei monopolarer Elektrochirurgie Neutralelektrode fern vom Device (>15 cm) platzieren, Stromfluss vom Device wegführend
- Defibrillator/Externe Pacing-Bereitschaft sicherstellen
- Prophylaktisches Aufkleben von Defibrillator-Pads in Hochrisikosituationen (Schrittmacher-abhängige Patientinnen und Patienten bei CIED-naher Chirurgie (<15 cm Abstand) oder Unzugänglichkeit des Gerätes sowie Risiko einer Impedanzerhöhung mit Exit-Block)
- Kommunikation mit OP-Team im präoperativen Team-Timeout über das Vorgehen bei intraoperativen CIED-Komplikationen

10. **Postoperative Kontrolle**

- Sofortige oder zumindest zeitnahe Nachkontrolle durch Kardiologie bei präoperativer Umprogrammierung. Kontinuierliche Überwachung am Monitor bis zur Kontrolle.
- Device-Kontrolle gemäß hausinternen Richtlinien.
- Unmittelbare oder zeitnah postoperative Kontrolle nach offensichtlichem Auftreten von EMI. Kontinuierliche Überwachung am Monitor sowie externe Pacing-/Defibrillationsbereitschaft bis zur Kontrolle.
